# Episodic Memory Impairment Mediates the Loss of Awareness in Mild Cognitive Impairment

**DOI:** 10.3389/fnagi.2021.802501

**Published:** 2022-01-21

**Authors:** Geoffroy Gagliardi, Patrizia Vannini

**Affiliations:** ^1^Neurology, Brigham and Women's Hospital, Boston, MA, United States; ^2^Neurology, Massachusetts General Hospital, Boston, MA, United States; ^3^Harvard Medical School, Cambridge, MA, United States

**Keywords:** awareness, episodic memory, executive functions, amyloid, Alzheimer's disease

## Abstract

**Introduction:**

Loss of awareness is a common symptom in Alzheimer's Disease (AD) and responsible for a significant loss of functional abilities. The mechanisms underlying loss of awareness in AD is unknown, although previous findings have implicated dysfunction of primary executive functioning (EF) or episodic memory (EM) to be the cause. Therefore, our main study objective was to explore the involvement of EF and EM dysfunction in amyloid-related loss of awareness across the clinical spectrum of AD.

**Methods:**

A total of 895 participants (362 clinically normal [CN], 422 people with mild cognitive impairment [MCI] and 111 with dementia) from the Alzheimer's Disease Neuroimaging Initiative were used for the analyses. A sub-analysis was performed in 202 participants who progressed in their clinical diagnosis from CN to MCI or MCI to dementia as well as dementia patients. Mediation models were used in each clinical group with awareness (assessed with the Everyday Cognitive function questionnaire) as a dependent variable to determine whether EF and/or EM would mediate the effect of amyloid on awareness. We also ran these analyses with subjective and informant complaints as dependent variables. Direct correlations between all variables were also performed.

**Results:**

We found evidence for a decline in awareness across the groups, with increased awareness observed in the CN group and decreased awareness observed in the MCI and dementia groups. Our results showed that EM, and not EF, partially mediated the relationship between amyloid and awareness such that greater amyloid and lower EM performance was associated with lower awareness. When analyzing each group separately, this finding was only observed in the MCI group and in the group containing progressors and dementia patients. When repeating the analyses for subjective and informant complaints separately, the results were replicated only for the informant's complaints.

**Discussion:**

Our results demonstrate that decline in EM and, to a lesser degree, EF, mediate the effect of amyloid on awareness. In line with previous studies demonstrating the development of anosognosia in the prodromal stage, our findings suggest that decreased awareness is the result of an inability for the participant to update his/her insight into his/her cognitive performance (i.e., demonstrating a petrified self).

## 1. Introduction

Alzheimer's Disease (AD) is characterized by a specific pattern of brain pathology and cognitive impairment sufficient to interfere with functional activities of daily living (ADL) (Sperling et al., 2011; Dubois et al., 2016; Jack et al., 2018). Within the past decades, research made it possible to detect some biomarkers associated to AD (e.g., using brain imaging, blood sample, or genetic analyses). Two pathological hallmarks of AD, amyloid and tau (Braak and Braak, 1991; Braak et al., 2006), have been shown to accumulate, following a topographic sequence that can be related to the cognitive phenotype (Bejanin et al., 2017). Numerous neuroimaging studies have demonstrated that the accumulation of these pathologies begins decades before cognitive decline, and hence before a clinical diagnosis of AD can be made (Jansen et al., 2015; Ossenkoppele et al., 2015). It has been argued that pathology, occurring as early as the preclinical stage, is the cause of subtle cognitive impairments (Amieva et al., 2008, 2014; Baker et al., 2017; Zhao et al., 2018) primarily in executive functioning (EF) and episodic memory (EM) (Amieva et al., 2008, 2014; Hedden et al., 2013; Zhao et al., 2018). Of these, EF is defined as an assembly of cognitive processes that allows the person to perform an untrained goal-directed task that may involve both planning and inhibition of automatic behaviors (Lezak, 1982; Norman and Shallice, 1986; Miyake et al., 2000; Godefroy et al., 2010). Biologically, EF has primarily been associated with the integrity of frontal regions (Godefroy et al., 2010; Bettcher et al., 2016; Guarino et al., 2019). In contrast, EM is defined as a person's capacity to acquire and recall information associated with a temporo-spatial, and potentially affective (although this later is not always necessary), context (Tulving, 1972, 1985; Eustache and Desgranges, 2008). Being one of the core clinical symptoms at the AD dementia stage (Dubois et al., 2014), EM dysfunction has been demonstrated to show a strong relationship with pathology, especially in the medial temporal lobe (MTL) regions (Bejanin et al., 2017; Maass et al., 2018; Lowe et al., 2019). Although both EF (Elias et al., 2000; Baudic et al., 2006; Amieva et al., 2008, 2014; Marshall et al., 2011) and EM (Elias et al., 2000; Grober et al., 2000; Grober, 2008; Hedden et al., 2013) are impaired early in AD, some studies suggest that impairments in EM are a better and/or earlier predictor of prospective AD (Binetti et al., 1996; Derby et al., 2013; Burnham et al., 2016; Schindler et al., 2017). Moreover, declining EM and EF have been shown to significantly impair activities of daily living (ADL) by themselves (Marshall et al., 2011), but decline in these processes have also been associated with secondary impact in other cognitive domains. Importantly, it has been argued that a decline in these two processes could have an impact on an individual's self-referential processing, i.e., the awareness of our own cognitive abilities (Hannesdottir and Morris, 2007). However, the cause of changes in self-awareness, and especially the involvement of EF and EM dysfunction in loss of awareness across the AD spectrum, is unknown.

The prevalence of patients demonstrating loss of awareness (a.k.a., anosognosia) has been shown to increase along with the clinical progression of AD, with reported rates of 20 to 80% at the dementia stage (Starkstein, 2014). First used to describe two patients who, after a stroke, were unaware of their hemiplegia (Babinski, 1914), the concept of unawareness is now being applied more broadly. That is, although the circumstances may vary (Orfei et al., 2008), anosognosia is often used to describe a lack of awareness for a cognitive, behavioral or functional impairment (Weiler et al., 2016; Mograbi and Morris, 2018). Moreover, previous studies have proposed the existence of two types of anosognosia (Hannesdottir and Morris, 2007): primary anosognosia, which is described as an impairment of metacognitive processes and the inability for the individual to build a representation of oneself, and secondary anosognosia, which is the consequence of a decline in either EF or EM. In the first case, individuals would fail to either recognize or take into account their failures, while in the latter they would not be able to maintain the memory of such failures. Both incapacities prevent the individual from updating his/her own representations of cognitive functioning. In the case of EM impairment, this has led to the notion of a “petrified self,” indicating that the individual is relying on outdated information to create this representation (Mograbi et al., 2009; Morris and Mograbi, 2013).

Although anosognosia is very common at the clinical stage of AD, recent studies have shown that awareness starts to change even before the AD dementia stage (Folstein et al., 1975; Cacciamani et al., 2017, 2020). Previous research has shown that some individuals may demonstrate heightened awareness of subtle cognitive changes at the preclinical stage, with a subsequent decline of awareness as the individual moves along the AD trajectory (Vannini et al., 2017a, 2020; Hanseeuw et al., 2020). Additionally, recent studies have suggested that loss of awareness may be present in the predementia stages of AD, e.g., in the prodromal (Perrotin et al., 2015; Vannini et al., 2017c; Edmonds et al., 2018; Munro et al., 2018; Therriault et al., 2018) and even preclinical stages (Folstein et al., 1975; Cacciamani et al., 2017; Vannini et al., 2017b). Longitudinal studies have further demonstrated that anosognosia starts to develop approximately 3-4 years before a clinical diagnosis of AD dementia can be made (Wilson et al., 2015; Hanseeuw et al., 2020). Furthermore, there is now evidence suggesting that loss of awareness is related to the accumulation of biomarkers of AD (Cacciamani et al., 2017; Gagliardi et al., 2020, 2021). Awareness has indeed been studied in relation to brain metabolism, functional connectivity, tau, and amyloid accumulation. Loss of awareness in AD has been shown to relate to brain hypometabolism (Starkstein, 2014), even at a preclinical stage (Cacciamani et al., 2017). Similarly, some authors have showed that anosognosia in AD could be associated with dysfunction of some brain regions (i.e., frontal and temporo-parietal), as well as the functional connectivity between these brain regions (Perrotin et al., 2015; Vannini et al., 2017b,c). In addition, a recent study showed that unawareness was related to tau burden—as measured with flortaucipir PET marker—in the medial temporal region (Gagliardi et al., 2021). Amyloid burden has significantly been found to be associated with changes in awareness over the course of AD, either through a phenomenon of heightened (Visser et al., 2009; Perrotin et al., 2012) or reduced (Cacciamani et al., 2017; Vannini et al., 2017a, 2020; Therriault et al., 2018; Gagliardi et al., 2020, 2021; Hanseeuw et al., 2020) awareness. This relationship with amyloid could indicate that loss of awareness might be specific to AD. Additionally, some authors also found a relationship between anosognosia and brain connectivity in the Default Mode Network (DMN) (Mondragón et al., 2019, 2021). It is important to note that some authors showed that amyloid and tau accumulation patterns in the brain overlapped with the DMN (Wang et al., 2013). This allows us to hypothesize that an increasing brain pathology in these regions might affect their functioning and lead to loss of awareness and other cognitive functions, such as EF and EM (Greicius et al., 2004; Hedden et al., 2009; Sheline et al., 2010; Mormino et al., 2011; Brier et al., 2012).

However, it is still not clear if and how a deficit in EM or EF is related to changes in awareness in AD. Specifically, it is unknown whether a deficit in EF or EM mediates the AD pathology-related changes in awareness in a population comprised of cognitively normal (CN) individuals, those with a mild cognitive impairment (MCI), or those with a dementia diagnosis. Several methods have been validated to assess awareness in AD (Clare et al., 2011; Starkstein, 2014; Tondelli et al., 2018), such as clinical judgment by an examiner or the comparison between a subjective and an external measure. One of the most commonly used methods is to compare the subject's perception of his/her impairment with the informant's perception (Cacciamani et al., 2017, 2020; Hanseeuw et al., 2020; Vannini et al., 2020). Loss of awareness would then be defined as the informant complaining more than the subject. Using this approach, the current study attempts to determine the proportion of amyloid-related loss of awareness that can be explained by is EF and EM variations along the clinical AD spectrum (from the preclinical to dementia stage). Following the concept of secondary anosognosia and considering the early changes of awareness, EF, and EM, we hypothesize that loss of awareness is—at least partly—mediated by impairment of these cognitive domains, even in early stages of the disease. Additionally, given the previous observations of a decline in awareness as the disease progresses, we hypothesize that a decrease in awareness would be the result of the informant's complaint increasing as the participant's objective performance decline. To explore this hypothesis, the same models as for awareness were applied separately to the complaints of both participants and their informants.

## 2. Materials and Methods

### 2.1. Population

Data used in the preparation of this article were obtained from the Alzheimer's Disease Neuroimaging Initiative (ADNI) database (adni.loni.usc.edu). The ADNI is an ongoing, longitudinal, multicenter study conducted at 59 sites across North America, enrolling CN, amnestic MCI, and AD participants aged 55 to 94 years. The ADNI was launched in 2003 as a public-private partnership, led by Principal Investigator Michael W. Weiner, MD. The primary goal of ADNI has been to test whether serial magnetic resonance imaging (MRI), PET, other biological markers, and clinical and neuropsychological assessments can be combined to measure the progression of MCI and early AD. For up-to-date information, see www.adni-info.org.

In total, 902 participants were included in this study. Inclusion criteria included having a positron emission tomography (PET) scan data using a ^18^*F*−*AV*45 tracer for brain amyloidosis, subjective and objective cognitive measures (see *infra*), as well as an available clinical diagnosis. Using the clinical diagnosis, we further subdivided the sample into 362 CN participants, 429 with MCI and 111 with a dementia diagnosis. Demographic characteristics are summarized in Table 1.

**Table 1:** Demographic data and group comparisons.

**Variables**	**All**	**CN**	**MCI**	**Dementia**
N	902	362	429	111
Gender	0 (0 %)	0 (0 %)	0 (0 %) a**	0 (0 %) a**
Race	829 White (91.91 %)	328 White (90.61 %)	401 White (93.47 %)	100 White (90.09 %)
Ethnicity	859 Not Hisp/Latino (95.23 %)	341 Not Hisp/Latino (94.2 %)	413 Not Hisp/Latino (96.27 %)	105 Not Hisp/Latino (94.59 %)
Age	72.28 (7.08)	72.17 (6.32)	71.73 (7.34)	74.81 (7.84) a** b**
Education (Years)	16.4 (2.58)	16.75 (2.52)	16.22 (2.61) a**	15.98 (2.58) a**
MMSE (/30)	27.92 (2.39)	29.08 (1.17)	28.17 (1.67) a**	23.21 (2.04) a** b**
TMT B-A (Time in s.)	66.87 (54.23)	47.08 (35.13)	66.9 (48.28) a**	131.34 (74.04) a** b**
Logical Memory (Delayed Recall)	8.97 (5.06)	13.34 (3.21)	7.25 (3.18) a**	1.35 (1.85) a** b**
Amyloid (AV45 PET SUVr)	1.19 (0.22)	1.11 (0.17)	1.21 (0.23) a**	1.39 (0.22) a** b**
ECog - Self	1.64 (0.51)	1.38 (0.33)	1.81 (0.53) a**	1.85 (0.54) a**
ECog - Informant	1.62 (0.68)	1.19 (0.28)	1.7 (0.59) a**	2.71 (0.63) a** b**
Awareness (S vs I)	0.02 (0.68)	0.19 (0.34)	0.11 (0.7) a*	-0.86 (0.79) a** b**

*CN = Cognitively Normal, MCI = Mild Cognitive Impairment, MMSE = Mini-Mental State Examination, TMT = Trail Making Test, ECog = Everyday Cognition questionnaire, PET = positron emission tomography, SUVr = standard uptake value ratio, a = vs. CN, b = vs. MCI = p < 0.07*,

* * = p < 0.05*,

** = p < 0.01, *** = p < 0.001.

We also defined a group of participants that could be considered to be in the Alzheimer spectrum, i.e., participants who progressed in their clinical diagnosis from CN to MCI (*N*= 27) or MCI to dementia (*N*= 64), as well as dementia patients (*N*= 111). This group will be referred to as the “progressors” group.

### 2.2. Cognitive Measures

All participants underwent a comprehensive neuropsychological assessment. Among the different tests available, we selected the Mini-Mental State Examination (MMSE) (Folstein et al., 1975), Trail Making Test (TMT) (Reitan, 1958), and Logical Memory (LM) (Wechsler, 1987). The MMSE was used to provide a measure of global cognitive functioning. The time difference of TMT parts A and B was used as a measure of EF, as done in previous research (Godefroy et al., 2010; Correia et al., 2015; Ritchie et al., 2017). This test is recommended for preclinical AD studies (Ritchie et al., 2017) and has been shown to distinguish between CN participants with and without significant levels of amyloid pathology (Doherty et al., 2015; Dubois et al., 2018). For EM, we used the delayed score of LM (LM-II), widely used in AD continuum research and proven to be sensitive, even at preclinical stages, to biomarker accumulation (Ritchie et al., 2017). Both tests are among the most-used measures in AD studies (Epelbaum et al., 2017) and have been used in previous studies examining awareness Starkstein (2014); Vannini et al. (2017a); Hanseeuw et al. (2020); Cacciamani et al. (2020); Gagliardi et al. (2020).

### 2.3. Awareness of memory

The Everyday cognition (ECog) scale (Farias et al., 2008) was used to assess awareness. The ECog scale is a 39-item questionnaire in which the participant and informant are asked identical questions to estimate the participant's current level of cognitive functioning as compared to 10 years ago. The responses are measured on a Likert scale from 1 (“Better or no change”) to 4 (“Consistently much Worse,”) where a higher ECog score indicates a perceived decline in cognition. The questionnaire consists of 6 domain-specific subscales, including Memory, Language, Visuospatial Abilities, Planning, Organization, and Divided Attention. A total score of cognitive complaint is also computed. In order to measure awareness, we computed an awareness index defined as the discrepancy score between the total scores of the participant and informant reports. Using this index, a negative score would indicate that the participant is overestimating his/her capacities as compared to the informant's appraisal (i.e., unawareness), whereas a positive score would indicate that the participant is underestimating his/her capacities as compared to the informant's appraisal (i.e., heightened awareness) (Vannini et al., 2017a, 2020; Hanseeuw et al., 2020). A score of 0 would indicate a perfect match between participant and informant complaints.

### 2.4. Imaging

Florbetapir (^18^*F*−*AV*45) PET tracer was used to measure brain amyloidosis. We used a global standard uptake value ratio (SUVr) computed using the whole cerebellum as a reference region. Amyloid was used as a continuous measure in all analyses. The details of the materials and methods related to florbetapir imaging have been described in Landau et colleagues (Landau et al., 2013).

### 2.5. Statistical Analyses

*T*-tests and chi-squared tests were used to compare continuous and categorical variables between the three clinical groups (i.e., CN, MCI, and Dementia). The relationship between our dependent and independent variables were explored using two main methods.

First, Pearson correlations were performed between awareness and TMT B-A, LM-II and amyloid within the whole sample and in the separate clinical groups. This analysis was performed to determine whether direct relationships between our dependent and independent variables existed in our whole sample and/or in our subgroups. This aimed to support the interpretation of potential relationships observed in the subsequent models. Second, linear regression models were also performed to explore the same relationships, taking into account demographic variables (see supplementary materials).

A model-based causal mediation analyses using a causal inference analysis approach (Imai et al., 2010) was used to investigate whether memory or executive function mediates the relationship between amyloid and awareness. Specifically, mediation analyses were performed on the whole sample, in the sub-group that contained progressors and dementia patients, and in each clinical group, using the awareness index as the dependent variable, amyloid as the independent variable, and cognition (either TMT B-A for EF, or LM-II for EM) as the mediator. Demographics (i.e., age, gender, and education) were included as covariates in all models. Both total, direct and mediated effects were calculated. Mediation models were computed using the mediation R package (Tingley et al., 2014). Standard errors were estimated using a quasi-Bayesian Monte-Carlo method based on normal approximation (Imai et al., 2010) with 1,000 iterations. All presented *p*-values were corrected for multiple comparisons using the Benjamin-Hochberg method. The models looking at the three clinical groups (CN, MCI, Dementia) were corrected for 9 comparisons (i.e., 3 groups and 3 conditions—awareness, informant, subject). The models looking at the Whole Sample and progressors group for 6 comparisons (2 groups and 3 conditions). All statistical analyses were performed using R 3.6.3 (https://www.R-project.org/).

## 3. Results

### 3.1. Group Comparisons

The sample consisted of 362 clinically normal and 540 clinically impaired individuals (429 of which had MCI diagnosis and 111 diagnosed with dementia; see Table 1). As compared to CN participants, clinically impaired participants demonstrated a lower proportion of females as well as fewer years of education (mean years; both *p < 0.01*). Although no significant difference was observed between CN and MCI groups, participants diagnosed with dementia were significantly older those in both the CN and MCI groups (both *p < 0.01*). Group comparisons revealed that CN individuals showed better cognitive performance than MCI participants who, in turn, performed better than the dementia group (all *p < 0.01*). Group comparisons further revealed that dementia patients demonstrated significantly increased amyloid burden as compared to MCI participants who, in turn, had significantly greater amyloid burden than CN participants (all *p < 0.01*). Regarding awareness, CN participants demonstrated greater levels of awareness as compared to the MCI group who, in turn, exhibited significantly higher scores than the dementia group (all *p < 0.01*). We observed that the informant complaints were at a lower level in the CN group as compared to MCI, and in the MCI group as compared to the dementia group (all *p < 0.01*). Finally, CN participants demonstrated lower subjective complaints than the MCI and the dementia groups (both *p < 0.01*). However, MCI participants and those diagnosed with dementia exhibited the same level of subjective complaints (*p > 0.05*).

### 3.2. Simple Correlations

**Figure 1:**
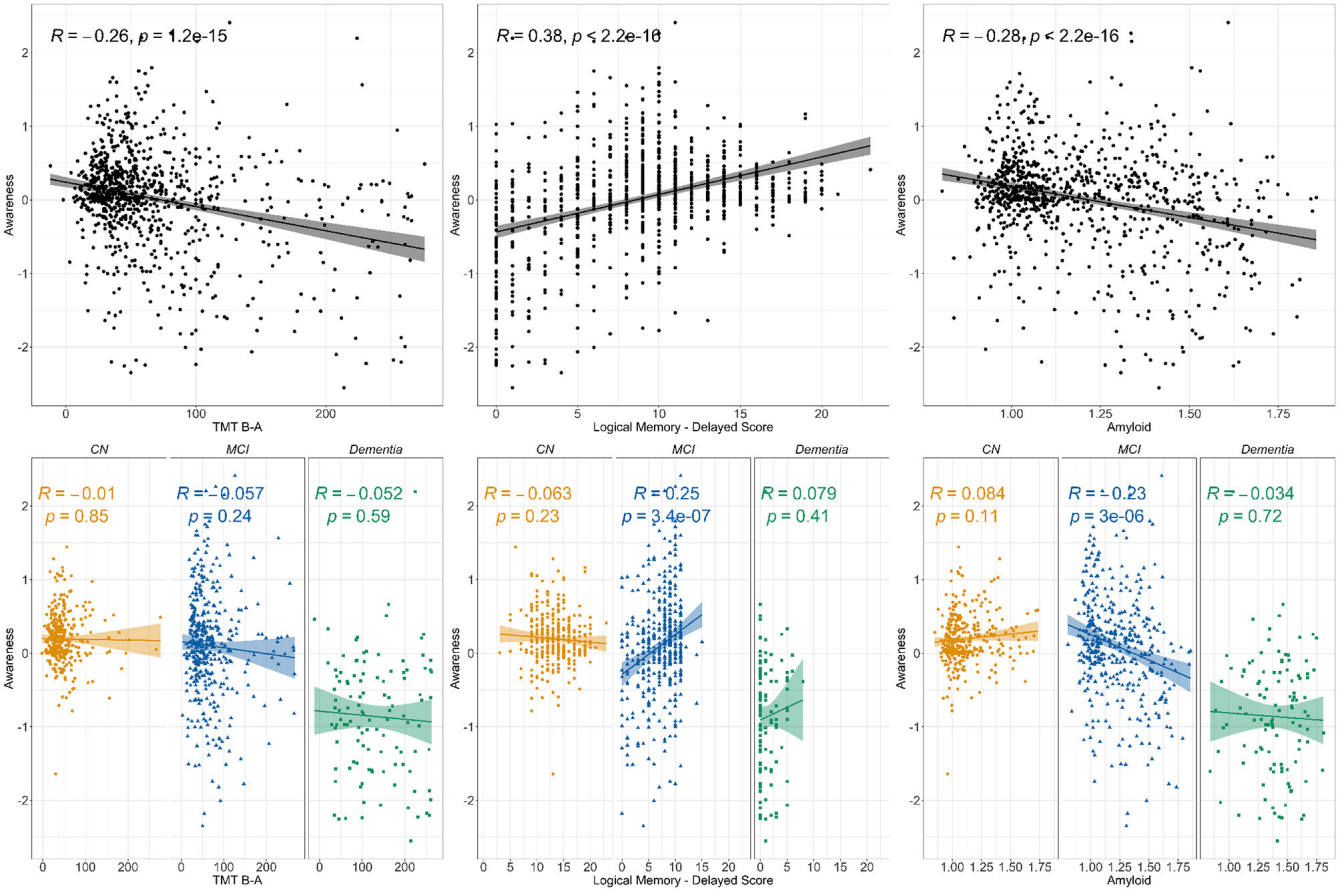
Correlations between awareness and TMT B-A, Logical memory delayed score and amyloid. *Notes:* The figure presents the Pearson correlation coefficient for the relationship between awareness and cognition (Trail Making Test B-A for executive function and Logical Memory's delayed recall for episodic memory) as well as amyloid (SUVr). The top line presents the correlations for the whole sample while the bottom line displays clinical groups correlations. Three clinical groups are represented, i.e., Cognitively Normal (CN), Mild Cognitive Impairment (MCI), and participants diagnosed with Dementia.

All correlations are presented in Figure 1. When using the whole sample, the correlation analyses revealed weak negative significant associations between awareness and TMT B-A as well as between awareness and amyloid SUVr. A moderate positive significant association was found between awareness and LM-II (all *p <0.001*). When looking at the groups separately, the same pattern was observed in the MCI group for LM-II (r = 0.25, *p <0.001*) and amyloid SUVr (r=-0.23, *p <0.001*), but non-significant relationships were observed in the other groups. No significant correlations were observed for TMT B-A in any of the clinical groups. The linear regression models showed similar results for EM and amyloid (see Supplementary Table and Supplementary Figures 2, 3). However, when adding demographics and diagnosis as covariates, TMT B-A was no longer significant in the whole sample (see Supplementary Table and Figure 1).

### 3.3. Mediation Models

Mediation models were performed to assess the influence of cognitive performance (episodic memory or executive function) on the relationship between amyloid and awareness, as well as the separate levels of complaint in informants and participants. In each model, the indirect effect was tested using bootstrapping procedures with 1,000 iterations.

#### 3.3.1. Mediation Models Investigating Whether EM or EF Mediates the Association Between Amyloid and Awareness

**Figure 2:**
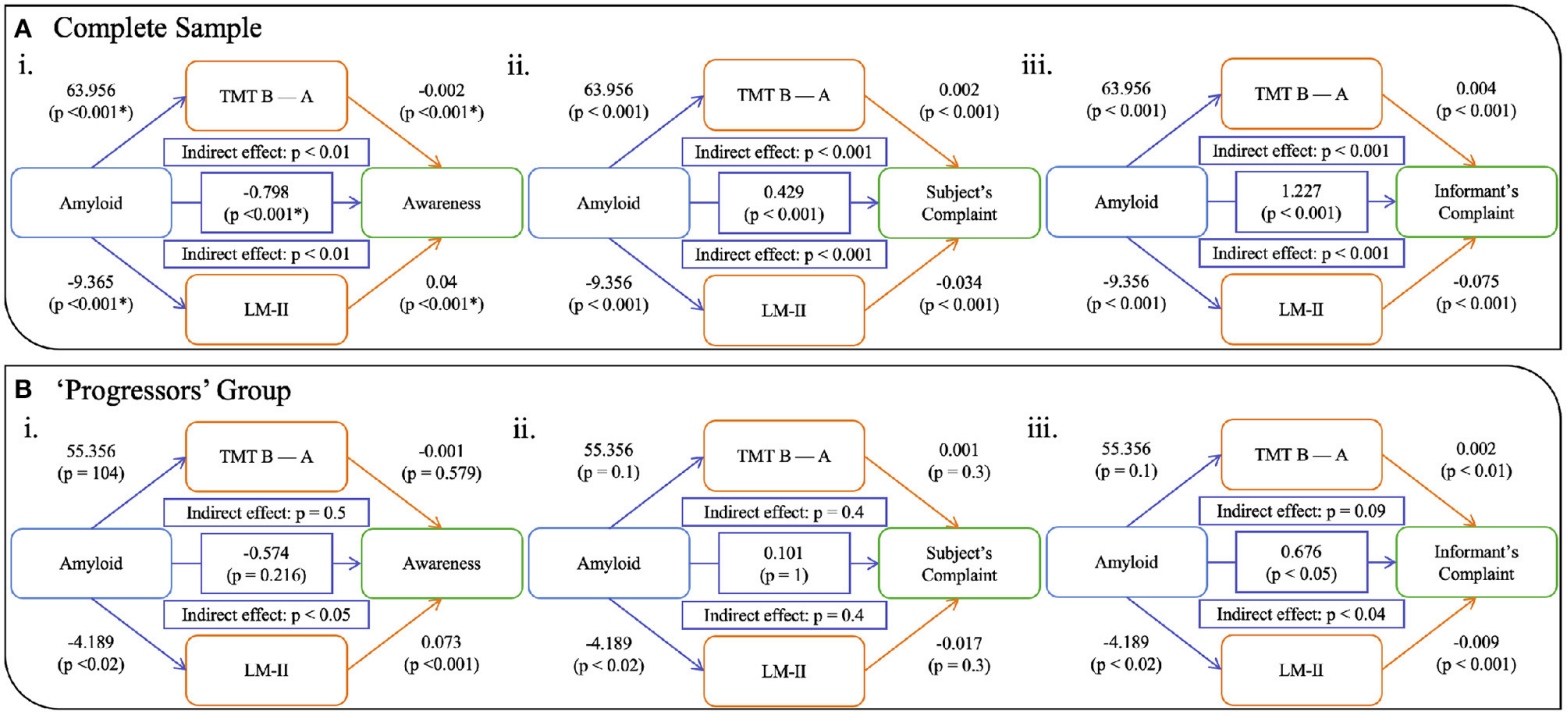
Models showing the mediation of EM and EF on the relationship between amyloid and awareness (i), subjective complaints (ii), and informant's complaints (iii) in **(A)** the whole sample and **(B)** the progressor and dementia group. *Notes:* TMT B-A = Trail Making Test, Time B minus Time A; LM-II = Logical Memory Delayed Recall. All *p*-values are corrected for multiple comparison.

**Figure 3:**
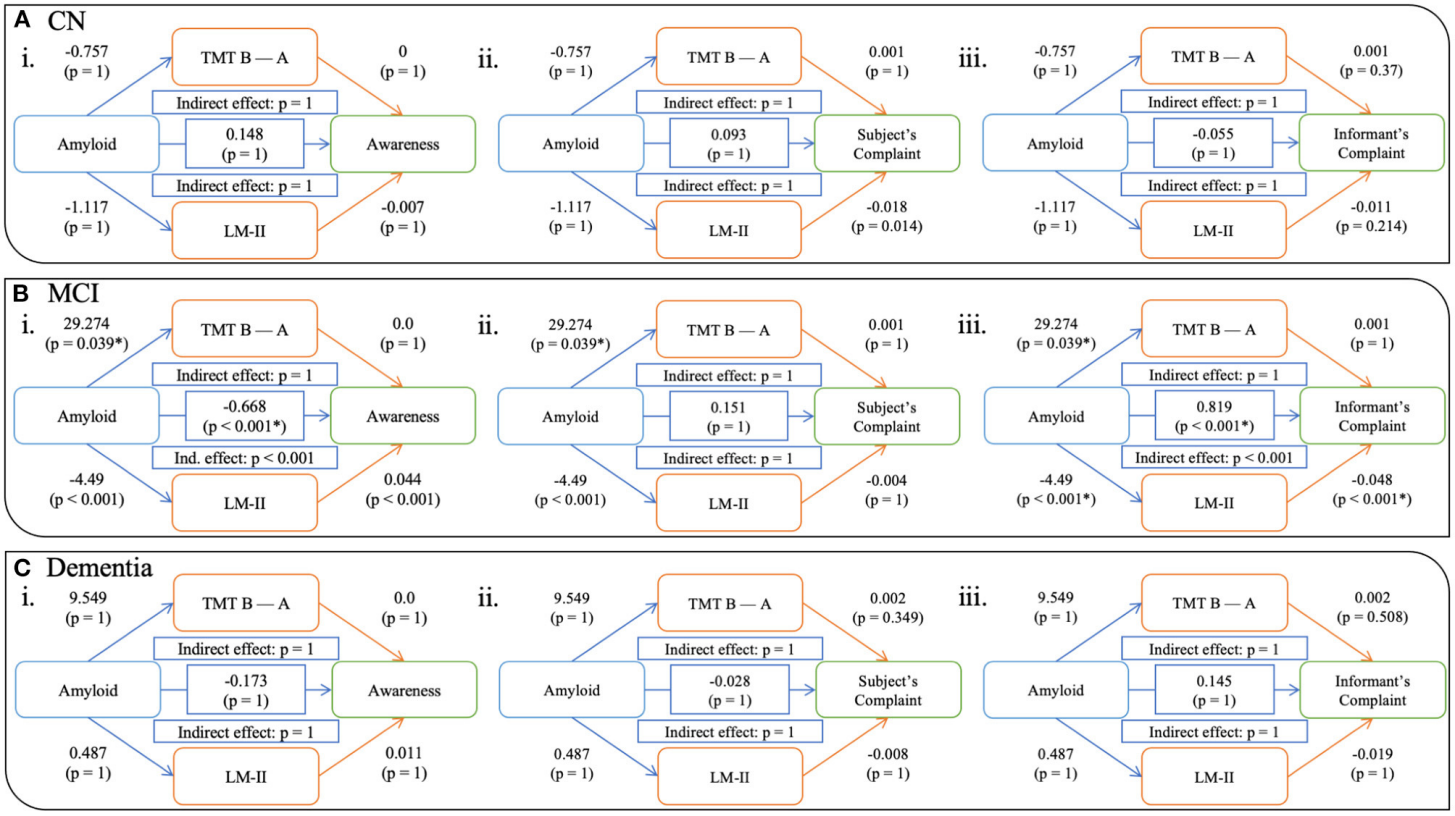
Models showing the mediation of EM and EF on the relationship between amyloid and awareness (i), subjective complaints (ii), and informant's complaints (iii) in CN **(A)**, MCI **(B)**, and dementia **(C)** participants. *Notes:* All *p*-values are corrected for multiple comparison. CN = Cognitively Normal, MCI = Mild Cognitive Impairment.

For the whole sample, the effect of amyloid on awareness was partially mediated by cognition—both EM and EF (Figure 2Ai). A significant direct effect between amyloid and awareness was observed (β= -0.798; CI 95%= -1, -0.57; *p < 0.001*) in that increased amyloid burden was related to a lower awareness score. Both EM and EF analyses demonstrated significant indirect effect (both *p < 0.001*), with the unstandardized indirect effect equaling -0.657 (CI 95%= 0.083, 0.325; *p < 0.001*) for TMT B-A and -0.421 (CI 95%= 0.338, 0.701; *p < 0.001*) for LM-II. While no significant direct effect of amyloid was observed on awareness, in the “progressors” group (Figure 2Bi), we found a significant indirect effect of EM (β= −0.27; CI 95% = 0.049, 2.664; *p > 0.05*), but not of EF (unstandardized indirect effect β= −0.499; CI 95% = −0.175, 0.886; *p > 0.05*). When looking at each clinical group separately, this pattern was not observed for all groups (see Figure 3i). That is, no significant relationships, either direct or indirect, were found between amyloid and awareness in the CN or Dementia groups. However, in the MCI group, we found that LM-II partially mediated the effect between amyloid and awareness. That is, similarly to in the whole sample, this model displayed a significant direct effect between amyloid and awareness (β= −0.668; CI 95%= −0.998, −0.349; *p < 0.001*) as well as a significant indirect effect mediated by the LM-II delayed score (β= −0.199; CI 95%= −0.351, −0.083; *p < 0.001*). No significant indirect effect was observed for TMT B-A.

#### 3.3.2. Mediation Models Investigating Whether EM or EF Mediates the Association Between Amyloid and Complaints

We also computed models using complaints from the participant (ii in Figures 2, 3) as well as their informant (iii in Figures 2, 3). Similar to the awareness index, results using the whole sample demonstrated partial mediation between amyloid and complaints (subjective and informant) with both executive and memory measures. Interestingly, when looking at these relationships in the groups separately, different patterns were observed. Models assessing participant complaints did not show any significant relationship, either direct or indirect, between amyloid and complaint after correction for multiple comparisons in any group (CN, MCI, Dementia, and “progressors”). Models predicting the informant's complaint in CN and Dementia participants did not show any significant associations. In the MCI group (Figure 3iii) a significant direct relationship was found such that a greater amyloid burden was related to increased informant complaints (β = 0.819; CI 95% = 0.572, 1.048; *p < 0.001*). No indirect effect was observed for executive functioning, although amyloid SUVr significantly predicted TMT B-A performance (β = 29.274, *p < 0.05*). However, a significant indirect effect was found for LM-II delayed score (β = 0.603; CI 95% = 0.341, 0.863; *p < 0.001*), indicating that it mediated the effect between amyloid and informant complaints in the MCI participants. The “progressors” group showed the same pattern as in the MCI model, with a significant direct effect of amyloid on the informant's complaint, as well as a significant indirect effect in EM (β = −0.27; CI 95% = 0.049, 2.664; *p < 0.05*) but not in EF (*p > 0.05*).

## 4. Discussion

The current study investigated whether episodic memory and/or executive function mediates the relationship between amyloid and awareness in individuals across the AD spectrum, from preclinical to dementia stages. In the whole group, episodic memory and executive function partially mediated the associations between amyloid and awareness, such that greater amyloidosis was related to less cognitive efficiency, which in turn negatively impacted awareness. However, when looking at each group separately, we could only observe this mediation effect in the MCI group. When looking at the subjective and informant complaints separately, we found that episodic memory partially mediated the associations between amyloid and informants' complaints, with greater amyloidosis being related to less cognitive efficiency and increased complaints by the informant. When analyzing the participants who either progressed or had a diagnosis of dementia, we also found that EM partially mediated the relationship between amyloid and awareness as well as informant complaints. All other mediation models were non-significant. These findings suggest that decreased awareness may be the result of an inability for the participant to update his/her insight of his/her cognitive performance (i.e., demonstrating a petrified self) and further suggests the impairment is happening in the prodromal stage. Furthermore, given the association with amyloid, these findings also suggest that this decline may be specific to AD.

The demographics varied across our three clinical groups. That is, the clinically normal group contained more females as compared to the impaired participants (i.e., MCI and Dementia groups). These results differ from the literature, as previous studies often report greater numbers of females in impaired samples (Dubois et al., 2016; Livingston et al., 2020). However, in line with previous studies, we found that our clinically impaired participants were less educated and older as compared to the clinically normal group. They also performed less well on cognitive tasks (with CN performing better than MCI and MCI performing better than the dementia group). Finally, dementia participants demonstrated higher amyloid burden as compared to MCI participants who, in turn, had higher amyloid burden as compared to the CN participants.

Looking at our awareness index, we found an increasing discordance between informant *vs*. participant complaint scores across the clinical stages. This suggests the progressive loss of awareness across the AD spectrum which has been shown in previous studies (Wilson et al., 2015; Hanseeuw et al., 2020). Looking more in detail, the awareness index was the highest in the CN group, suggesting heightened awareness, whereas it declined in the MCI group and further dropped in the Dementia group. These results could be interpreted in the sense that mean anosognosia appears progressively over the course of the disease and is preceded by a period of heightened awareness (or hyper-nosognosia) in the earlier stages (Folstein et al., 1975; Cacciamani et al., 2017; Vannini et al., 2017a, 2020; Hanseeuw et al., 2020). However, as the disease progresses, individuals progressively lose their ability to recognize their cognitive difficulties, ultimately resulting in low insight of their cognitive processing, often at the prodromal stage. Although the mechanism underlying the loss of awareness remains unknown, one hypothesis suggests that it is caused by the fact that the participant is relying on an outdated representation of their own abilities when judging their own cognitive performance, a phenomenon known as the “*petrified-self* ” (Mograbi et al., 2009). In longitudinal studies this can be demonstrated by a non-significant increase in subjective complaints over time, even though their cognitive processes are declining, and their informant complaints are increasing.

Regarding the relationships between awareness and our variables of interest, we found that decreased awareness was related to increased amyloid burden, as well as lower EM and EF performances. These relationships should be interpreted with the disease progression in mind. That is, amyloid is thought of as one of the main and first biomarkers to accumulate in AD (Sperling et al., 2011; Dubois et al., 2016; Jack et al., 2018), even decades before a clinical diagnosis is made (Jansen et al., 2015; Ossenkoppele et al., 2015). Previous studies have shown that amyloid often accumulates in the ventromedial prefrontal cortex, medial parietal and posterior cingulate cortex, as well as the inferior parietal lobule. Interestingly, these brain regions overlap with the DMN (Wang et al., 2013), which has been implicated in self-referential processes (Mak et al., 2017) as well as EF and EM functioning (Greicius et al., 2004; Hedden et al., 2009; Sheline et al., 2010; Mormino et al., 2011; Brier et al., 2012). Within this framework, one possibility is that alterations in memory self-awareness represent an early indicator of progressive decline toward AD dementia due to increased dysfunction of the DMN. This hypothesis is consistent with previous studies relating loss of awareness in AD with DMN connectivity (Mondragón et al., 2019, 2021), but also with the relationship between anosognosia in AD with some neuropsychiatric disorders (Spalletta et al., 2012; Tondelli et al., 2021) which have also been associated with dysfunction of the DMN (Lee et al., 2020). When looking at the groups separately, we only observed a significant correlation between awareness and EM in the MCI individuals. The same pattern was found when looking at the mediation analyses, with partial mediation effects for the whole sample (for the awareness index as well as both complaints) for both EM and EF models. These results suggest that the significant effect that we observed of amyloidosis on awareness is mediated by an individual's objective performance of either EM or EF. In other words, amyloid accumulation in the brain would causes a decline in EM and EF that leads to a disturbance in awareness. However, when looking at each group separately, the results were not as clear. First, for all groups, the models using self-complaints did not show any significant relationship between amyloidosis and complaints, neither direct nor indirect. This result is not in line with previous studies which have found an association between increased amyloid pathology with increased complaints in clinically normal individuals (Jessen et al., 2010; Amariglio et al., 2012; Wang et al., 2013; Perrotin et al., 2015), a phenomenon called subjective cognitive decline (SCD). However, inconsistencies have been reported and might be due to the fact that SCD itself can be caused by multiple etiologies (Jessen et al., 2014, 2020; Rabin et al., 2017). In addition, some models using awareness and informant complaints remained significant for our groups. That is, although models in the CN and the Dementia groups did not show significant effects, neither direct nor indirect, significant partial mediations were observed in the MCI group with EM. To begin with, increased discordance between the informant's and participant's complaints have already been observed in the literature, using raw complaint scores (Amariglio et al., 2021) as well as awareness indices as in our study (Folstein et al., 1975; Cacciamani et al., 2017, 2020; Hanseeuw et al., 2020; Vannini et al., 2020). In our study, only the MCI group demonstrated a significant relationship between amyloidosis, cognition and awareness, with EM partially mediating the relationship between amyloid and awareness. Several reasons could explain this result. First, this might be a power issue, as the MCI group included more participants than the other groups. Another more plausible reason might be related to the dynamic of awareness changing across time. Previous research has proposed that awareness changes in the early stages of AD, with an initial increase in awareness before a subsequent decline, eventually leading to anosognosia in the prodromal and/or dementia stage (Vannini et al., 2017a). However, it is unclear when heightened awareness can be observed, with some studies suggesting it can be found in the preclinical (Folstein et al., 1975; Cacciamani et al., 2017; Hanseeuw et al., 2020) stage while other argue that it is observed in the prodromal (Vannini et al., 2017c; Edmonds et al., 2018; Munro et al., 2018; Therriault et al., 2018; Tondelli et al., 2018) stage of the disease. Accordingly, we could hypothesize different scenarios that might explain the non-significant effect found in the CN and Dementia groups. That is, in the preclinical stage, we might assume that the subtle cognitive decline that participants are experiencing is not sufficient for the effect to be detectable using objective neuropsychological tests, i.e., the EM and EF may be showing a ceiling effect. In contrast, in the dementia group, the lack of relationship between cognition and awareness might be since all measures might be too declined, i.e., having reached a floor effect. Another interpretation of the absence of a significant mediation of EM/EF on the relationship between amyloid and awareness in the dementia group could be that as unawareness progresses, anosognosia in the dementia stage might be the cause of primary anosognosia and not secondary anosognosia. Finally, the absence of an effect in the CN group can be explained by the fact that this group likely is heterogeneous, including both normal and preclinical participants. The absence of an effect could thus be explained by this mixture, and perhaps an early effect could be detected if future progressors and stable CN individuals were separated in a larger sample and follow-up (i.e., as our sample only included 27 CN progressors).

It is important to note that, in the MCI group, only the EM mediated effect survived. In the typical AD phenotype, EM has been demonstrated to be the cognitive domain that is most affected (Dubois et al., 2014). As previously mentioned, amyloidosis accumulates following a pattern that would disturb regions subserving EM. Previous studies have furthermore showed that EM starts to decline years before the clinical diagnosis of AD dementia (Elias et al., 2000; Grober, 2008; Derby et al., 2013), but would initially be too tenuous to be detectable in a regular neuropsychological assessment at the preclinical stage. In the literature, unawareness has been proposed to be due to either a primary dysfunction of awareness processes, or a secondary effect due to impairment in either EM or EF processes (Hannesdottir and Morris, 2007). As compared to both CN participants, participants in the MCI roup demonstrated lower levels of awareness, as well as a lower cognitive efficiency (for both EM and EF). These results suggests that initial EM decline (i.e., at the preclinical stage / for CN participants) would not be sufficient to affect an individual's perception of growing difficulties. Participants would thus have correct insight into their initial memory decline. However, with the progression of this EM deficits, awareness would also start to show subsequent impairments. This scenario is also supported by our sub-analyses in the group containing progressors and dementia patients. Similar to the results observed in the MCI participants, we observed partial mediation of amyloid by EM on both awareness and informant complaints. These results suggest that the relationship between a decline in awareness and EM dysfunction is specific to AD. It also demonstrates the importance of the informant's reports as the disease progresses.

Additionally, the significant results in the group of participants that progressed can also be interpreted in another way. This “progressors” group consisted of both patients diagnosed with dementia or CN/MCI progressing to MCI/dementia. However, the mediation models showed significant results for the MCI group and not for the CN/dementia groups. Given the high proportion of MCI in this group, one possibility is that these individuals drove the results for this analysis.

The absence of a relationship (both in correlation and in the models) between awareness and the EF measure in the groups could be interpreted in several ways. In our study, we used the TMT B-A as a measure of EF functioning. However, EF is a generic term that refers to an ensemble of different high level cognitive processes (Lezak, 1982; Norman and Shallice, 1986; Miyake et al., 2000). The TMT, widely used as a measure of EF, has been suggested to involve working memory, switching and dividing attention (Strauss et al., 2006; Godefroy et al., 2010; Correia et al., 2015). Hannesdottir and Morris's definition suggested that this process involves a comparison executive mechanism (Hannesdottir and Morris, 2007) such that error detection, as well as correction, are part of EF (Luria, 1966). This, in turn, would rely partly on an attention orientation mechanism. Nevertheless, it is possible that the absence of a significant interaction involving the TMT could be because the task does not test specific executive processes that may be related to awareness. This could explain the discrepancy between our results and previous studies that found a relationship between EF and impaired awareness (Amanzio et al., 2013). For example, Amanzio and colleagues (Amanzio et al., 2013) did find such relationships using a dysexecutive battery including several tests, among which the TMT was included.

### 4.1. Limitations

This study has several limitations. To begin with, the TMT B-A was the only available measure to assess EF. In awareness conceptualization, executive dysfunctions that are postulated to be related to unawareness are comparison mechanisms (Hannesdottir and Morris, 2007). Thereby, it is possible that our models fail to show a relationship involving EF because the TMT could not involve the same type of EF processes as those suggested in the model. Future studies should investigate this further by using measures directly related to comparison and judgement. Another limitation is that we only used the global SUVr value when analyzing amyloid burden. Another approach could have been to focus on regional pathology, focusing specifically on brain regions known to be involved in awareness dysfunction. In particular, future studies should investigate whether increased amyloid in specific brain regions show an association with awareness and whether this differs between the groups. However, this approach might face the limit of multiple modelisation in order to be pursued. Further investigation also needs to be conducted using other pathological biomarkers, especially brain metabolism or tauopathy that are known to be more related to the cognitive phenotype (with the topography of the pathology matching the clinical expression) (Bejanin et al., 2017). Finally, a substantial number of studies have shown that AD can have a prevalence, expression and evolution that can vary depending on certain demographics. Our study suffers from an over-representation of Caucasian (91.91%), non-Hispanic (95.23%), and highly educated (16.41 mean years) participants. This limits the generalizability of our results and calls for future studies that replicate these findings in other, more diverse cohorts.

### 4.2. Conclustion

In summary, here, we studied the influence of both EF and EM on the relationship between amyloidosis and awareness across the AD spectrum. Our results suggest that EM, and not EF, mediates the effect between amyloid and awareness. In particular, a decrease of awareness was found in the MCI group and in participants that progressed to AD. This effect was not seen in the CN and Dementia stages, which might be explained by the fact that cognitive decline was either too subtle (in the CN group) or too advanced (in the dementia group) for the relationship to be detected.

## Data Availability Statement

Publicly available datasets were analyzed in this study. This data can be found at: http://adni.loni.usc.edu.

## Ethics Statement

The studies involving human participants were reviewed and approved by ADNI, obtained all IRB approvals and met all ethical standards in the collection of data. The following are the Ethics Committees and IRB boards that provided approval. The Ethics Committees/Institutional Review Boards that approved the ADNI study are: Albany Medical Center Committee on Research Involving Human Subjects Institutional Review Board, Boston University Medical Campus and Boston Medical Center Institutional Review Board, Butler Hospital Institutional Review Board, Cleveland Clinic Institutional Review Board, Columbia University Medical Center Institutional Review Board, Duke University Health System Institutional Review Board, Emory Institutional Review Board, Georgetown University Institutional Review Board, Health Sciences Institutional Review Board, Houston Methodist Institutional Review Board, Howard University Office of Regulatory Research Compliance, Icahn School of Medicine at Mount Sinai Program for the Protection of Human Subjects, Indiana University Institutional Review Board, Institutional Review Board of Baylor College of Medicine, Jewish General Hospital Research Ethics Board, Johns Hopkins Medicine Institutional Review Board, Lifespan - Rhode Island Hospital Institutional Review Board, Mayo Clinic Institutional Review Board, Mount Sinai Medical Center Institutional Review Board, Nathan Kline Institute for Psychiatric Research &amp; Rockland Psychiatric Center Institutional Review Board, New York University Langone Medical Center School of Medicine Institutional Review Board, Northwestern University Institutional Review Board, Oregon Health and Science University Institutional Review Board, Partners Human Research Committee Research Ethics, Board Sunnybrook Health Sciences Centre, Roper St. Francis Healthcare Institutional Review Board, Rush University Medical Center Institutional Review Board, St. Joseph's Phoenix Institutional Review Board, Stanford Institutional Review Board, The Ohio State University Institutional Review Board, University Hospitals Cleveland Medical Center Institutional Review Board, University of Alabama Office of the IRB, University of British Columbia Research Ethics Board, University of California Davis Institutional Review Board Administration, University of California Los Angeles Office of the Human Research Protection Program, University of California San Diego Human Research Protections Program, University of California San Francisco Human Research Protection Program, University of Iowa Institutional Review Board, University of Kansas Medical Center Human Subjects Committee, University of Kentucky Medical Institutional Review Board, University of Michigan Medical School Institutional Review Board, University of Pennsylvania Institutional Review Board, University of Pittsburgh Institutional Review Board, University of Rochester Research Subjects Review Board, University of South Florida Institutional Review Board, University of Southern, California Institutional Review Board, UT Southwestern Institution Review Board, VA Long Beach Healthcare System Institutional Review Board, Vanderbilt University Medical Center Institutional Review Board, Wake Forest School of Medicine Institutional Review Board, Washington University School of Medicine Institutional Review Board, Western Institutional Review Board, Western University Health Sciences Research Ethics Board, and Yale University Institutional Review Board. The patients/participants provided their written informed consent to participate in this study.

## Author Contributions

All authors contributed to the analyses, discussion of content, writing, reviewing and editing of the paper and approved the final version.

## Funding

Data collection and sharing for this project was funded by the Alzheimer's Disease Neuroimaging Initiative (ADNI) (National Institutes of Health Grant U01 AG024904) and DOD ADNI (Department of Defense award number W81XWH-12-2-0012). ADNI is funded by the National Institute on Aging, the National Institute of Biomedical Imaging and Bioengineering, and through generous contributions from the following: AbbVie, Alzheimer's Association; Alzheimer's Drug Discovery Foundation; Araclon Biotech; BioClinica, Inc.; Biogen; Bristol-Myers Squibb Company; CereSpir, Inc.; Cogstate; Eisai Inc.; Elan Pharmaceuticals, Inc.; Eli Lilly and Company; EuroImmun; F. Hoffmann-La Roche Ltd and its affiliated company Genentech, Inc.; Fujirebio; GE Healthcare; IXICO Ltd.; Janssen Alzheimer Immunotherapy Research &amp; Development, LLC.; Johnson &amp; Johnson Pharmaceutical Research &amp; Development LLC.; Lumosity; Lundbeck; Merck &amp; Co., Inc.; Meso Scale Diagnostics, LLC.; NeuroRx Research; Neurotrack Technologies; Novartis Pharmaceuticals Corporation; Pfizer Inc.; Piramal Imaging; Servier; Takeda Pharmaceutical Company; and Transition Therapeutics. The Canadian Institutes of Health Research is providing funds to support ADNI clinical sites in Canada. Private sector contributions are facilitated by the Foundation for the National Institutes of Health (www.fnih.org). The grantee organization is the Northern California Institute for Research and Education, and the study is coordinated by the Alzheimer's Therapeutic Research Institute at the University of Southern California. ADNI data are disseminated by the Laboratory for Neuro Imaging at the University of Southern California. This work has been funded by NIH-NIA R01 AG061083 (PI: PV) and NIH/NIA R21 AG064348 (PI: PV).

## Conflict of Interest

The authors declare that the research was conducted in the absence of any commercial or financial relationships that could be construed as a potential conflict of interest.

## Publisher's Note

All claims expressed in this article are solely those of the authors and do not necessarily represent those of their affiliated organizations, or those of the publisher, the editors and the reviewers. Any product that may be evaluated in this article, or claim that may be made by its manufacturer, is not guaranteed or endorsed by the publisher.

## Supplementary Material

Supplementary Table 1 Relationship between executive function measure and awareness.

Supplementary Table 2 Relationship between episodic memory measure and awareness.

Supplementary Table 3 Relationship between amyloid measure and awareness.

Supplementary Table 4 Number of participants extracted from ADNI cohorts.

Supplementary Figure 1 Relationship between executive function measure and awareness.

Supplementary Figure 2 Relationship between episodic memory measure and awareness.

Supplementary Figure 3 Relationship between amyloid measure and awareness.
